# Response of grassland net primary productivity to dry and wet climatic events in four grassland types in Inner Mongolia

**DOI:** 10.1002/pei3.10064

**Published:** 2021-10-07

**Authors:** Md Lokman Hossain, Md Humayain Kabir, Mst Umme Salma Nila, Ashik Rubaiyat

**Affiliations:** ^1^ Department of Environment Protection Technology German University Bangladesh Gazipur Bangladesh; ^2^ Department of Geography Hong Kong Baptist University Hong Kong; ^3^ Institute of Forestry and Environmental Sciences University of Chittagong Chittagong Bangladesh; ^4^ Wegener Center for Climate and Global Change University of Graz Graz Austria; ^5^ CEN Centre for Earth System Research and Sustainability Institute of Geography University of Hamburg Hamburg Germany; ^6^ Burckhardt Institute, Tropical Silviculture and Forest Ecology, Faculty of Forest Sciences and Forest Ecology University of Göttingen Göttingen Germany

**Keywords:** climate extremes, drought, grassland type, MODIS NPP, net primary productivity, SPEI, time‐lag effects

## Abstract

Increasing frequency and intensity of climate extremes have profound impacts on grassland biodiversity functioning and stability. Using Moderate Resolution Imaging Spectroradiometer (MODIS) net primary productivity (NPP) data and standardized precipitation evapotranspiration index, we assessed the response of NPP to growing‐season and annual climate extremes and time‐lag of climatic conditions across four grassland types (meadow steppe, typical steppe, steppe desert, and desert steppe) in Inner Mongolia, China from the period 2000 to 2019. Results showed that annual NPP varied significantly across four grassland types, with the highest NPP in meadow steppe and the lowest in desert steppe. Annual NPP of all grassland types increased over the past 20 years, but NPP in meadow steppe and typical steppe decreased for the period 2012–2019. Irrespective of grassland type, the 1‐ and 2‐month time‐lag of climatic conditions showed significant effects on annual NPP. Growing‐season climate was found the better predictor of annual NPP in all grassland types than the annual climate. Compared with growing‐season normal climates, annual NPP was lowest in extreme dry events in all grasslands, while highest in extreme wet events in meadow steppe and typical steppe, and in moderate wet events in steppe desert and desert steppe. Typical steppe and steppe desert are highly vulnerable to the increasing intensity of climate extremes, as we found that the losses of NPP in these grasslands in extreme dry were almost double than that of moderate dry events. Surprisingly, for meadow steppe and desert steppe, the losses of NPP for both moderate and extreme dry events were almost the same, which highlights that a low‐intensity drought may have profound impacts on the annual NPP of these grasslands. The study provides the key insight in scientific basis to improve our understanding of the effects of climate extremes on grassland NPP, which is critical to sustainable management of grassland and maintain ecosystem stability.

## 1 BACKGROUND

Grassland is the largest terrestrial ecosystem (Lecain et al., 2002), which makes up over 30% of earth terrestrial surface (Adams et al., 1990) and sequestrates about 30% of the total carbon of the terrestrial ecosystem (Kemp et al., 2013; Zhou et al., 2018). Grassland ecosystems provide numerous goods (e.g., food, fiber, fuel) and services (e.g., conservation of soil and water, control of soil erosion, purify the air) (Allan et al., 2015). Climate change is causing a great threat to grassland biodiversity (IPCC, 2013). The predicted changes in global climate are likely to have a major effect of the functioning and stability of ecosystems (Dong et al., 2021; Nila et al., 2019). Given the potential threat of global change to grassland biodiversity, understanding the response of grassland net primary productivity (NPP) to climate extremes (e.g., droughts) is a crucial challenge (Vicente‐Serrano et al., 2013), as numerous studies provided evidence that grassland has a higher susceptibility to climate extremes than other ecosystems (Li et al., 2018; Liu, Lin, et al., 2021; Zhang et al., 2017).

NPP is defined as the gross primary productivity of plants minus autotrophic respiration, which includes aboveground and belowground biomass (Roxburgh et al., 2005). NPP, an organic substance produced by photosynthesis, is the energy source of primary consumer and a key carbon cycle mechanism between terrestrial ecosystems and the atmosphere (Sun et al., 2016). As grassland is an important sink of global carbon, a small disturbance in the structure and the function of this ecosystem may have profound impacts on terrestrial carbon balance (Lei & Peters, 2003). Although numerous studies have shown NPP in many grasslands have been affected by grazing (Liang et al., 2018), fire (van der Werf et al., 2010), and land‐use change (Houghton & Goodale, 2004), recent studies reported that droughts have severe impacts on grassland NPP (Bao et al., 2019; Lei et al., 2020; Zhang, Miao, et al., 2020).

The growing trend in the frequency and intensity (e.g., mild, moderate, and extreme) of climate extremes have been found to affect grassland productivity (Bao et al., 2019; Wilcox et al., 2017). Many empirical evidence suggests that the response of aboveground NPP (ANPP) and belowground NPP (BNPP) to climate extremes vary greatly (Luo et al., 2017; Zhang, Cadotte, et al., 2019). For example, irrespective of grassland types, the effects of extreme wet climates on ANPP was either positive (Wilcox et al., 2017), negative (Padilla et al., 2019), or insignificant (Zhang, Cadotte, et al., 2019), and the effects of extreme dry climates on BNPP was either positive (Liu, Lin, et al., 2021), negative (Luo et al., 2017), or insignificant (Xu et al., 2015). Despite the great efforts that have been made to investigate the effects of climate extremes on grassland ANPP or BNPP, no consensus of the effects of climate extremes on grassland NPP has been achieved, since results showed a decrease of ANPP with climatic variability may enhance BNPP and vice‐versa (Dai et al., 2019; Hossain & Li, 2021a; Quan et al., 2020). For example, the effects of droughts on grassland productivity have been found positive in an African savanna, prairies of North America, and steppe in Ireland (Scott et al., 2010), negative in the desert steppe, typical steppe and meadow steppe in Inner Mongolia (Lei et al., 2020), and stable in experimental grassland in Germany (Kreyling et al., 2008). These disparate findings may be due to the variations of drought index classification and differences in grassland types. Many studies have used differential experimental droughts, such as 100% rainfall reduction for a specific period in growing‐season (Kreyling et al., 2017) and 30‐day rain‐free period (Li et al., 2020) were considered extreme dry events. While other studies have used standardized precipitation evapotranspiration index (SPEI) values <−1.3 (Barnes et al., 2016), standardized precipitation index (SPI) values ≤−2.0 (Lei et al., 2020), Palmer Drought Severity Index (PDSI) values <−4.0 (Wang et al., 2019) in order to classify extreme dry events. This differential climate event classifications in various grasslands may provide variations in the study findings. Thus, there is a need to use a globally consistent drought index classification in order to examine the effects of climate extremes on the grassland NPP.

Remote sensing data provides an advanced way to monitor ecosystem health (John et al., 2018). In recent years, the application of satellite‐derived Moderate Resolution Imaging Spectroradiometer (MODIS) NPP product to track vegetation dynamics and evaluate the relationships with abiotic factors have been widely used (de Leeuw et al., 2019; Xu et al., 2007; Zhao et al., 2014), as studies found strong agreement of field‐based measurement with MODIS NPP product (Turner et al., 2005, 2006). Although early attempts showed that the association between grassland NPP and climatic variability are complicated due to the variations in grassland types, the underlying heterogeneity can be explained by the application of a globally consistent drought index classification (Vicente‐Serrano et al., 2013). Several drought‐indices (e.g., scPDSI, SPI, SPEI) have been used to classify climate extreme intensity and direction across grassland ecosystems (Barnes et al., 2016; Cui et al., 2021; Lei et al., 2020; Wang et al., 2019), in which SPEI has been found to be more appropriate over other indices, as SPEI can distinguish water surplus and deficits conditions over longer time scales (e.g., 1–48 months, Vicente‐Serrano et al., 2012).

The scientific novelty of this study relies on the consideration of (i) both growing‐season and annual climatic conditions in investigating the response of annual NPP to three intensities (normal, moderate, and extreme) and three directions (wet, normal, and dry) of growing‐season and annual climatic events, (ii) time‐lag (1‐ to 4‐month) effects on annual NPP, and (iii) a globally consistent climatic event classification based on SPEI values over the past century. In this study, using MODIS NPP product with a spatial resolution of 500 m and SPEI values for the period 2000–2019, the responses of NPP to climate extremes in four grassland types (meadow steppe, typical steppe, steppe desert, and desert steppe) in Inner Mongolia, China were assessed. We further examined the loss of NPP caused by climate extreme intensity (i.e., moderate and extreme dry) in respective grassland types. Herein, the objectives of this study are (i) to assess the spatial variation of annual NPP across grasslands and evaluate the temporal pattern of NPP in respective grassland type, (ii) to investigate the response of annual NPP to the different intensities and directions of growing‐season and annual climate extremes, and the time‐lag of climatic conditions, and (iii) to estimate NPP loss under moderate and extreme dry events in four grasslands.

## 2 MATERIALS AND METHODS

### 2.1 Description of study area

We selected four grassland types in the Inner Mongolia (112°21’–118°22′E, 43°25′–47°33′N), an autonomous region of China, which includes meadow steppe, typical steppe, steppe desert, and desert steppe. Recent studies have demonstrated that these grasslands are of great threat of climatic variability (John et al., 2018; Wang et al., 2020). Vegetation of each grassland show clear transitions from others by various dominant species. For example, meadow steppe is dominated by *Leymus chinensis*, *Poa attenuata*, *Stipa baicalensis*, *and Festucca lenensis*, and typical steppe is mostly occupied by *Stipa grandis*, *Stipa krylovii*, *Leymus chinenis*, *and Carex duriusula*. Steppe desert and desert steppe are characterized by xerophyte herbs (e.g., *Stipa gobica*, *Stipa glareosa*), perennial grasses (e.g., *Allium polyrrhizum*), and xerophytic shrubs (e.g., *Artemisa xerophytica* and *Caragana sinica*) (John et al., 2016, 2018; Wang et al., 2020). Vegetation in meadow steppe and typical steppe are herbaceous in nature and shallow rooted species that are less tolerant to increasing climatic variability (John et al., 2018), while vegetation in steppe desert and desert steppe are deep rooted species that can absorb shocks resulted from disturbance (e.g., low precipitation in growing‐season) (Wang et al., 2020). Mean annual temperature is −2.2°C in meadow steppe, 3.0°C in typical steppe, 5.1°C in steppe desert, and 7.2°C in desert steppe (Wang et al., 2020). Annual precipitation shows a decreasing trend in the order of meadow steppe (350–500 mm)>typical steppe (300–400 mm)>steppe desert (135–311 mm)>desert steppe (45–215 mm) (Wang et al., 2020).

### 2.2 Data sources

NPP data were obtained from one of the MODIS products of the gap‐filled MOD17A3HGF‐Version 6 (Running & Zhao, 2019, available at https://doi.org/10.5067/MODIS/MOD17A3HGF.006). SPEI data were extracted from the SPEIbase v2.5 dataset developed based on the CRU 3.24.01 precipitation and potential evapotranspiration (Vicente‐Serrano et al., 2010, http://spei.csic.es/database.html).

### 2.3 Data processing

Raster images of study points were collected through one of the MODIS access data tools. The cell value of the raster has been extracted by using ArcGIS version 10.1. To make it easy to manage, the raster was projected to WGS 1984 and converted to tiff from hdf. Here, the Identify feature of ArcGIS is used to get the Pixel value. The pixel size of the raster of MODIS NPP product is 500 m and the temporal extent is 2000–2019 (Running & Zhao, 2019). The valid range for NPP data is −30,000 to 32,700 and the scale factor is 0.0001. The scale factor was applied with the pixel value to get the real NPP. The real value (kg C m^−2^ year^−1^) of NPP of each year was calculated by multiplying the valid value by the scale factor. Quality control label for each cell makes this data cleaned up version of MOD17 products by removing unsatisfactory inputs from the 8‐day leaf area index and the fraction of photosynthetically active radiation. The extracted real NPP values were then grouped based on grassland types. In order to validate the data, MODIS NPP of meadow steppe, typical steppe, and steppe desert was correlated with the observed NPP for the year 2018 (Figure S2). Although validation of remote sensing vegetation data with field‐measured values is important, the MODIS NPP data of desert steppe was not validated due to lack of field observation data of this grassland. However, using MODIS data, a recent study in Inner Mongolia has reported no overestimation or underestimation of extracted aboveground biomass values relative to the observed values (John et al., 2018). Given the strong correlation of MODIS and observed NPP in three grassland types, we assumed that MODIS NPP of desert steppe is also suitable for examination of NPP response to climate extremes.

SPEI has been widely used to calculate short term (e.g., 1‐, 2‐, 3‐month) to long term (e.g., 24‐, 36‐, 48‐month) drought conditions of a particular location. We used 3‐ and 12‐month SPEI values to identify the growing‐season and annual climate extremes, as both growing‐season and annual climates have been found strong influence on vegetation dynamics across different grassland types (Hossain & Li, 2020; Isbell et al., 2015; Tian et al., 2017). The 3‐ and 12‐month SPEI values represent the wet, normal, and dry conditions of the growing‐season and annual climates. We categorized the SPEI values into 5‐class drought classification (extreme dry, moderate dry, normal, moderate wet, and extreme wet; Table S1; Isbell et al., 2015). In this classification, normal climate SPEI values are between >−0.67 and <0.67 and climate extreme SPEI ranges between ≤−0.67 and ≥0.67 (see Table S1). This is a widely used drought index classification to identify and quantify the intensity of climate extremes (Isbell et al., 2015; Vicente‐Serrano et al., 2013; Zhang, Ameca, et al., 2019). As time‐lag of climate has been found significantly affect vegetation functioning (Zhe & Zhang, 2021), we also used 1‐, 2‐, 3‐, and 4‐month time‐lag of climatic conditions in order to identify which time‐lag of climatic conditions has strong effects on annual NPP of our studied grasslands.

In order to quantify the changes of NPP induced by moderate and extreme dry climates, NPP loss was calculated from the difference between the mean NPP of drought years and NPP of normal years, as shown in Equations (1) and (2) (Lei et al., 2015).

(1) $$\Delta {\text{NPP}}_{\;\mathrm{mod}\;}={\text{NPP}}_{\text{mean}}‐{\text{NPP}}_{\text{moderatedry}}$$

(2) $$\Delta {\text{NPP}}_{\text{exd}}={\text{NPP}}_{\text{mean}}‐{\text{NPP}}_{\text{extreme dry}}$$

In Equations (1 and 2), *∆*NPP_mod_ and *∆*NPP_exd_ represent NPP loss resulted from moderate and extreme dry events, NPP_mean_ indicates the long‐term average NPP across all normal years (i.e., NPP for the SPEI values between >−0.67 and <0.67), NPP_moderate dry_ represents NPP in moderate dry years (i.e., NPP for the SPEI values between >−1.28 and ≤−0.67), and NPP_extreme dry_ represents NPP in extreme dry years (i.e., NPP for the SPEI values ≤−1.28).

### 2.4 Data analysis

Using boxplots, NPP variations among the grassland types were obtained. First, the significance of the differences in the mean NPP between the grassland types was examined using a one‐way ANOVA. Second, a post‐hoc Tukey's honestly significant difference (HSD) test was performed to investigate the pairwise comparisons of NPP between grassland types, provided the significance of the differences in the mean NPP. Finally, using Kendall's correlation coefficient, temporal variations in NPP in the respective grassland type were obtained. Here, NPP was the dependent variable and the year was the independent variable. In order to evaluate the association between NPP and SPEI, and NPP and time‐lag at <.05 significance (*p*) level, the Pearson correlation (*R*) analysis was used. Here, NPP was the dependent variable and SPEI and time‐lag was the independent variable. The significance of the differences in the mean NPP among five climate extreme intensities was obtained using a one‐way ANOVA. Given the significance of the differences in NPP among climate extreme intensities, a post‐hoc Tukey's HSD test was performed in order to examine the pairwise comparison of NPP difference between grassland types. NPP loss caused by moderate and extreme dry events in respective grassland types were plotted using “ggplot.” All statistical analysis was done in the statistical package R version 4.0.3 (R Core Team, 2020).

## 3 RESULTS

### 3.1 Variations of annual NPP across grasslands

**FIGURE 1:**
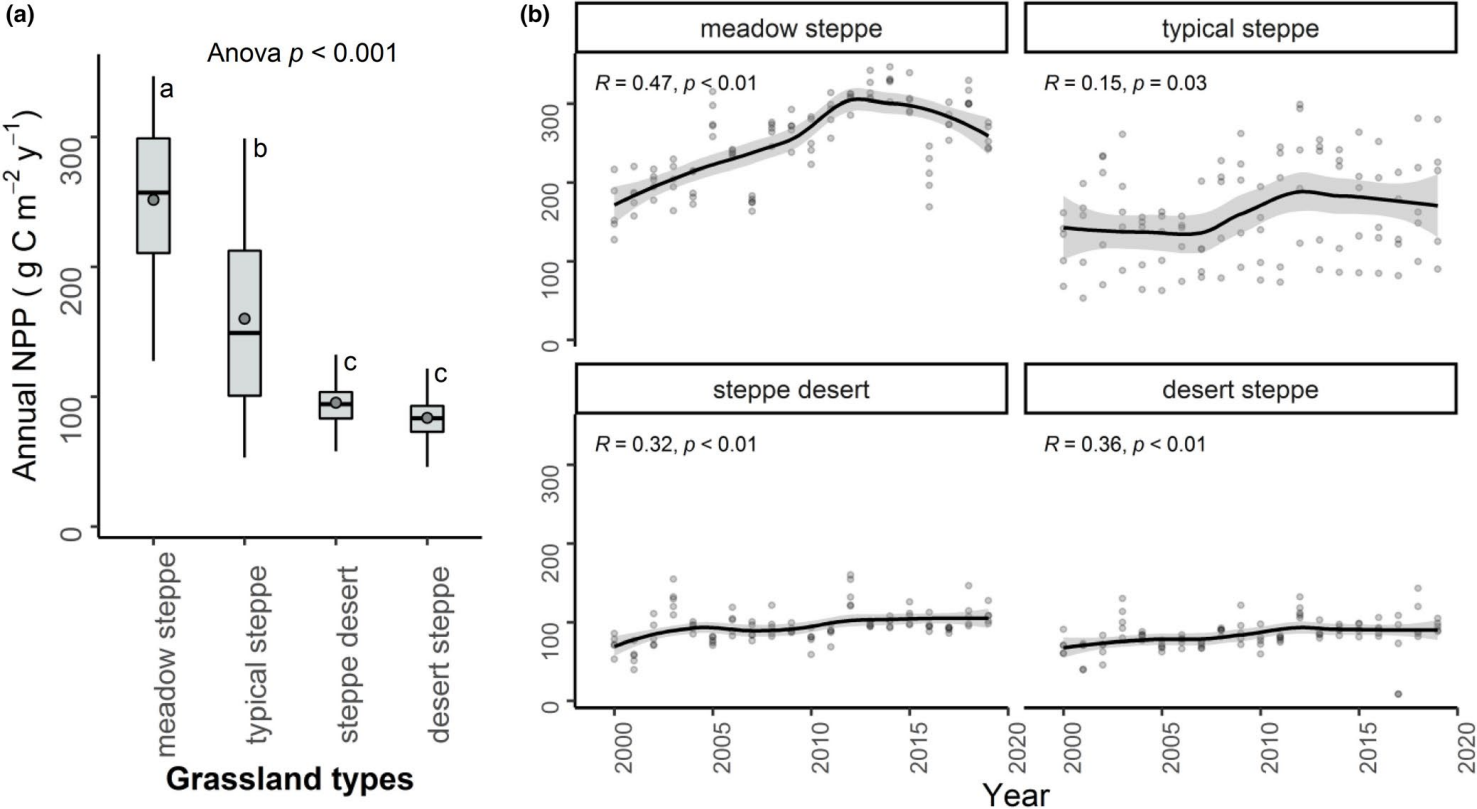
Spatial (a) and inter‐annual (b) variations of annual net primary productivity (NPP) (g C m^−2 ^year^−1^) across four grassland types during the period 2000–2019. ANOVA *p* in the boxplot indicates a significant difference in the mean NPP among four grassland types. Different letters (i.e., a, b, or c) on the top of the boxes indicate significantly different annual NPP among the grassland types at *p* < .05 in post‐hoc Tukey's HSD test. Whiskers in the boxes indicate the 95% confidence intervals of annual NPP in selected locations of each grassland type over 20 years. Solid horizontal lines in the boxes indicate the medians, circles in the middle of the boxes denote the mean NPP values, and boxes represent the first and third quartile. In inter‐annual variations of annual NPP (b), each point represents mean NPP value in each raster in respective grassland type. Smooth lines represent linear regressions of changes of NPP over the past 20 years. Bands near the lines indicate 95% confidence intervals of changes in annual NPP in selected locations of each grassland type during the period 2000–2019. Changes of NPP have been shown using Kendall's correlation coefficient (*R* and *p*)

The annual NPP exhibited large variations among the four grassland types (Figure 1a). NPP varied significantly among the grasslands (ANOVA *p* < .001), of which the mean NPP values were the highest in meadow steppe (251.65 gC m^−2 ^year^−1^) and the lowest in desert steppe (83.55 gC m^−2 ^year^−1^). Pairwise comparisons showed that NPP in all four grasslands differed significantly (Figure 1a, Figure S3, Table S2, all *p* < .001), except between the NPP values of steppe desert and desert steppe (Table S2, *p* > .05).

Although inter‐annual variations in NPP in meadow steppe and typical steppe showed positive trends (meadow steppe: *R* = .47, *p* < .01; typical steppe: *R* = .15, *p* = .03), NPP in these two grasslands showed decreasing trends for the year 2012–2019 (Figure 1b). Whereas, the increasing trends of NPP were consistent in steppe desert (*R* = .32, *p* < .01) and desert steppe (*R* = .36, *p* < .01) for the period 2000–2019 (Figure 1b).

### 3.2 Relationships between NPP and SPEI and time‐lag

**FIGURE 2:**
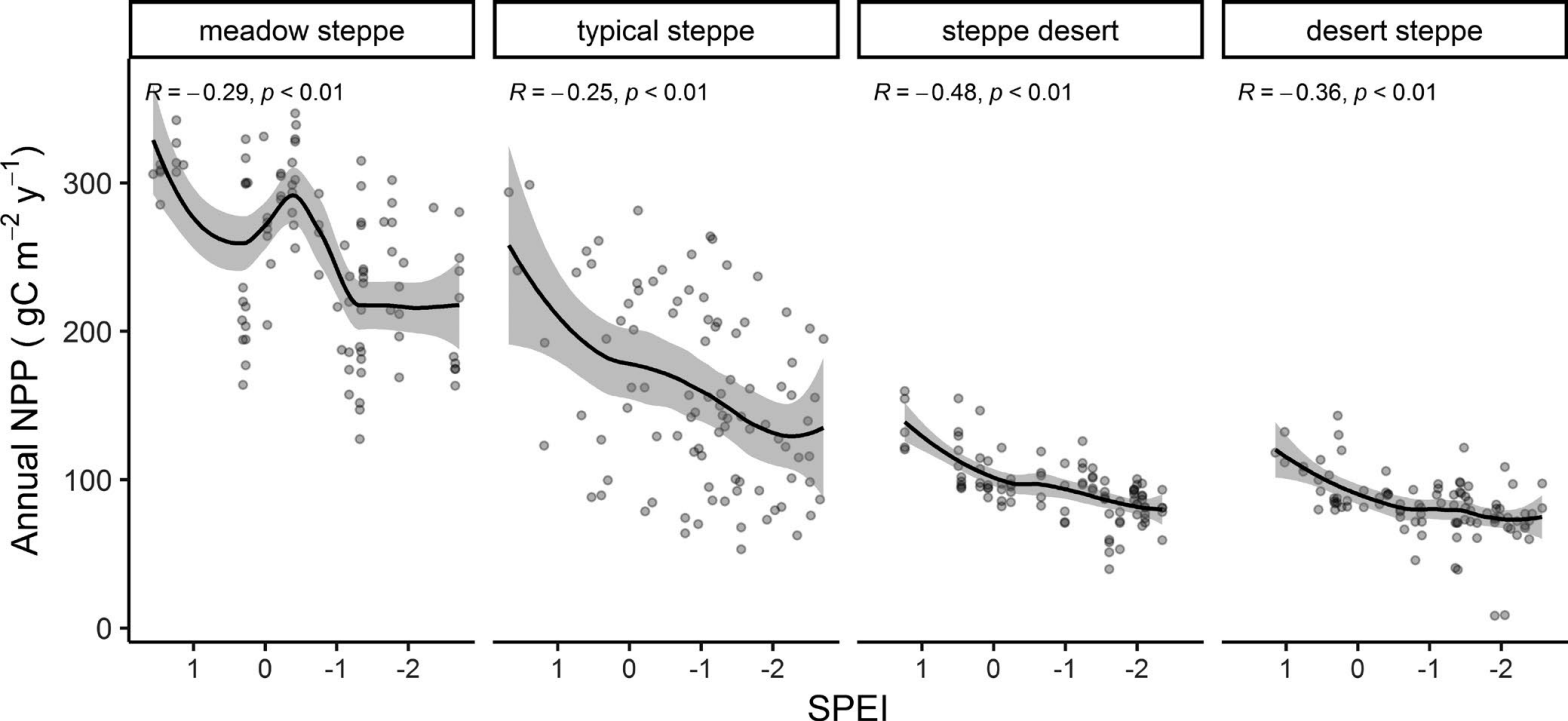
The correlation between annual net primary productivity (NPP) and growing‐season SPEI in four grassland types. Smooth lines represent linear regressions of the correlations between annual NPP and growing‐season SPEI. Bands near the lines represent 95% confidence intervals of the correlations between annual NPP and growing‐season SPEI. Pearson's correlation coefficient values (*R* and *p*) are shown

The annual NPP showed decreasing trends with increasing dry climates (i.e., decrease of SPEI values) for all grassland types (Figure 2, meadow steppe: *R* = –.47, *p* < .001; typical steppe: *R* = –.40, *p* < .001; steppe desert: *R* = –.65, *p* < .001; desert steppe: *R* = –.50, *p* < .001). Like the correlation between annual NPP and growing‐season SPEI, a significant correlation between NPP and 1‐month time‐lag (Table 1, all *p* < .001; meadow steppe: *R* = −.53; typical steppe: *R* = −.52; steppe desert: *R* = −.55; desert steppe: *R* = −.49) and 2‐month time‐lag (Table 1, all *p* < .01; meadow steppe: *R* = −.37; typical steppe: *R* = −.41; steppe desert: *R* = −.29; desert steppe: *R* = −.30) of climate was also observed in all grassland types. The relationship between NPP and other time‐lags (i.e., 3‐ and 4‐month) of climate were not significant (all *p* > .05), except for the 3‐month time‐lag of climate in meadow steppe (*R* = −.18, *p* < .05, Table 1) and typical steppe (*R* = −.22, *p* < .05, Table 1). Given the large variations of NPP with SPEI, the SPEI values were categorized into five climate event intensities in order to detect the changes of annual NPP in respective intensities (i.e., extreme wet, moderate wet, normal, moderate dry, and extreme dry) of growing‐season and annual climates.

**TABLE 1:** Correlation coefficients (*R*) between annual NPP and time‐lag of climatic conditions

Time‐lag	Grassland type			
	Meadow steppe	Typical steppe	Steppe desert	Desert steppe
1‐Month time‐lag	−.53***	−.52***	−.55***	−.49***
2‐Month time‐lag	−.37**	−.41**	−.29**	−.30**
3‐Month time‐lag	−.18*	−.22*	−.12	−.11
4‐Month time‐lag	−.09	−.06	−.03	−.13

* *p* < .05

** *p* < .01

*** *p* < .001.

### 3.3 Response of NPP to climate event intensity

**FIGURE 3:**
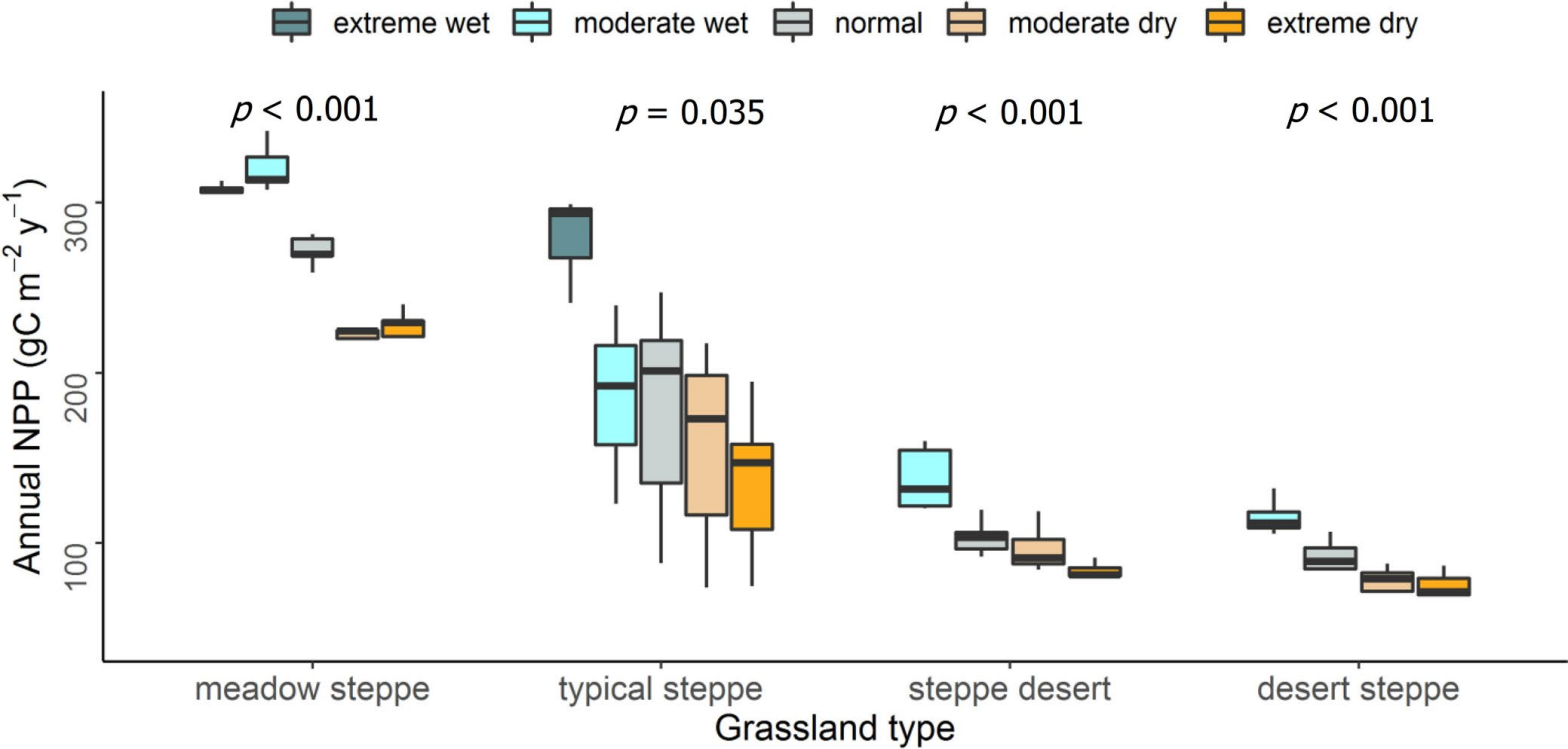
The response of annual net primary productivity (NPP) to growing‐season climate event intensities (extreme wet, moderate wet, normal, moderate dry, and extreme dry) in four grassland types (i.e., meadow steppe, typical steppe, steppe desert, and desert steppe). ANOVA *p* indicates a significant difference in the mean NPP among the growing‐season climate event intensities in respective grassland type. Boxes represent the first and third quartiles, solid horizontal lines in the boxes are the medians, and whiskers in the boxes denote the 95% confidence intervals of annual NPP response to each climatic event

One‐way ANOVA results showed that annual NPP varied significantly among the growing‐season climate event intensities for all grassland types (Figure 3, all ANOVA *p* < .05). Compared with growing‐season normal climates, growing‐season wet events showed higher NPP while growing‐season dry events showed lower NPP for all grassland types (Figure 3). In meadow steppe, the lowest NPP was recorded in growing‐season extreme dry events (225.15 gC m^−2 ^year^−1^), and the highest was in growing‐season moderate wet events (320.42 gC m^−2^year^−1^). Similarly, for typical steppe, the highest NPP was observed in growing‐season extreme wet events (277.87 gC m^−2^ year^−1^) and the lowest was in growing‐season extreme dry events (136.51 gC m^−2 ^year^−1^). No extreme wet events were observed in steppe desert and desert steppe (Figure 3). For both these grasslands, growing‐season moderate wet events exhibited the highest NPP (137.68 gC m^−2 ^year^−1^ in steppe desert, and 115.26 gC m^−2 ^year^−1^ in desert steppe), while growing‐season dry events had the lowest NPP (82.26 gC m^−2 ^year^−1^ in steppe desert in growing‐season extreme dry, and 73.24 gC m^−2 ^year^−1^ in desert steppe during growing‐season moderate dry climates). No significant differences in the mean annual NPP were observed among the annual climate event intensities (Figure S4), except significant difference of annual NPP between extreme dry and extreme wet climates (Figure S5) in steppe desert grassland (Figure S4, ANOVA *p* < .05).

**FIGURE 4:**
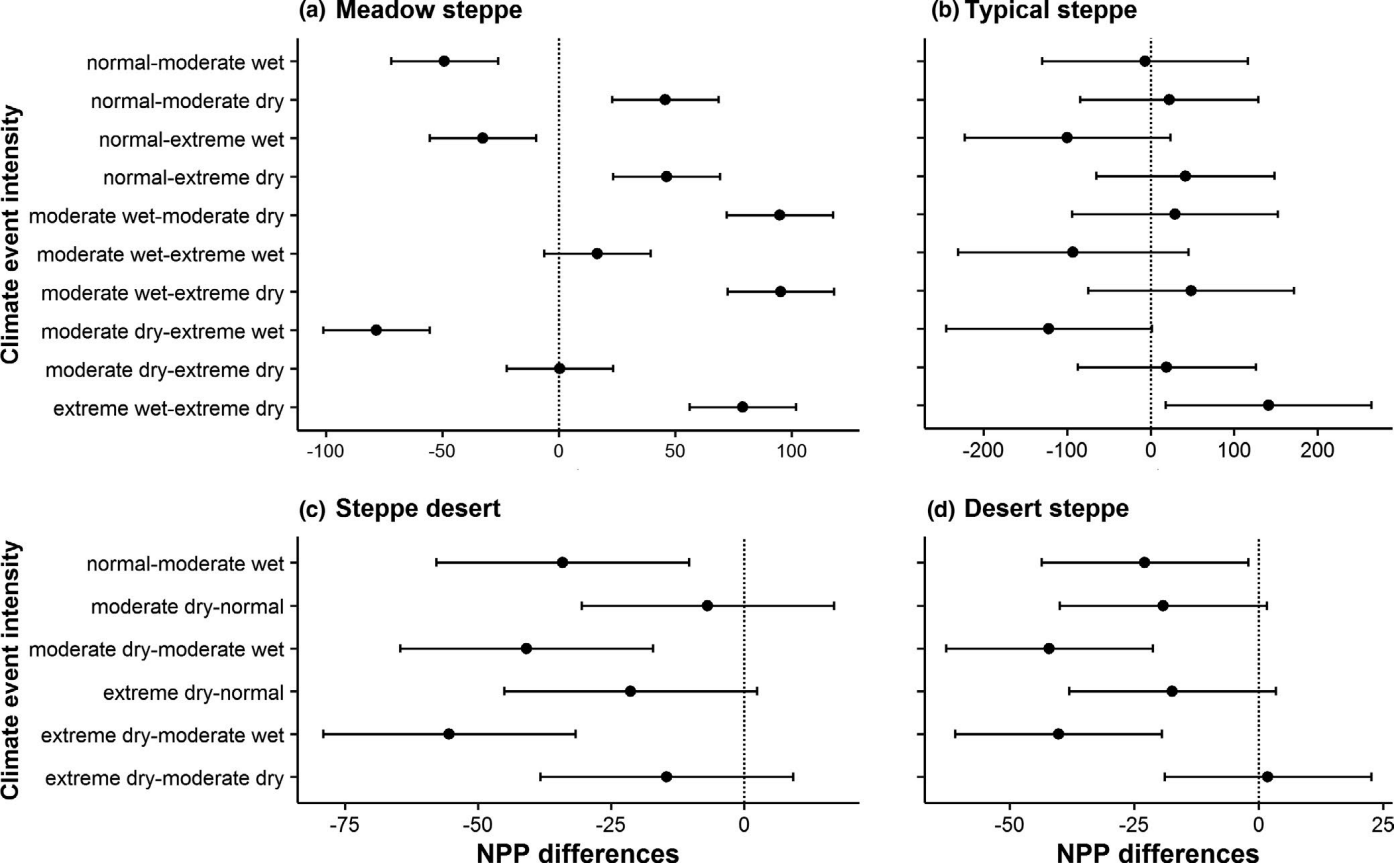
Pairwise comparisons of net primary productivity (NPP) values between the growing‐season climate event intensities for meadow steppe (a), typical steppe (b), steppe desert (c), and desert steppe (d). NPP differences were observed with post‐hoc Tukey's HSD test. The confidence intervals that do not contain 0 represent the significant difference in the pairs. The *p* values of the multiple pairwise comparisons of NPP are shown in Table 2

Pairwise comparisons of NPP values between the climate event intensities revealed that NPP in meadow steppe differed significantly between all intensities in growing‐season climates (Figure 4; Table 2a, all *p* < .01), except between moderate wet and extreme wet, and between moderate dry and extreme dry events (Figure 4; Table 2a, all *p* > .05). For the typical steppe, NPP values significantly differed for the pairwise comparisons of growing‐season extreme wet and extreme dry (Figure 4; Table 2b, *p* = .02) and of growing‐season moderate dry and extreme wet (Figure 4; Table 2b, *p* = .05), while the pairwise comparisons between the NPP of other growing‐season climate event intensities were not significant (Figure 4; Table 2b, all *p* > .05). Surprisingly, for the steppe desert and desert steppe, the differences of NPP between normal and dry events (i.e., normal‐moderate dry and normal‐extreme dry) were not significant (Figure 4; steppe desert: Table 2c, all *p* > .05, and desert steppe: Table 2d, all *p* > .05). In these two grasslands, significantly higher NPP was observed in growing‐season moderate wet events in comparison with normal, moderate dry, and extreme dry events (Figure 4; Table 2c,d, all *p* < .05). As steppe desert and desert steppe did not experience growing‐season extreme wet events (i.e., no growing‐season showed SPEI values ≥1.28) during the study period, no pairwise comparisons between extreme wet and other events were obtained (Figure 4; Table 2c,d).

**TABLE 2:** Pairwise comparisons of NPP values between the growing‐season climate event intensities for meadow steppe (a), typical steppe (b), steppe desert (c), and desert steppe (d). The NPP differences between each pair were given and the corresponding *p* values of the comparisons were obtained using post‐hoc Tukey's HSD test

Climate event intensity	(a) Meadow steppe		(b) Typical steppe		(c) Steppe desert		(d) Desert steppe	
	NPP difference	*p* value	NPP difference	*p* value	NPP difference	*p* value	NPP difference	*p* value
Extreme wet‐extreme dry	78.82	**<.001** ***	141.35	.**020** *	NA	NA	NA	NA
Moderate dry‐extreme dry	0.44	.991	19.22	.980	14.52	.332	−1.83	.994
Moderate wet‐extreme dry	95.26	**<.001** ***	48.45	.749	55.42	**<.001** ***	40.17	**<.001** ***
Normal‐extreme dry	46.18	**<.001** ***	41.65	.754	21.32	.086	17.31	.119
Moderate dry‐extreme wet	−78.38	**<.001** ***	−122.13	.**05** *	NA	NA	NA	NA
Moderate wet‐extreme wet	16.44	.237	−92.90	.282	NA	NA	NA	NA
Normal‐extreme wet	−32.63	.**003** **	−99.70	.145	NA	NA	NA	NA
Moderate wet‐moderate dry	94.82	**<.001** ***	29.23	.947	40.90	**<.001** ***	42.01	**<.001** ***
Normal‐moderate dry	45.74	**<.001** ***	22.42	.965	6.80	.844	19.15	.075
Normal‐moderate wet	−49.07	**<.001** ***	−6.80	.999	−34.09	.**004** **	−22.85	.**028** *

The significance level (5%, 1%, and .1%) of NPP difference between climate event intensities has been shown in bold values (*p* < .05, *p* < .01, and *p* < .001).

Abbreviation: NA, not applicable.

* *p* < .05

** *p* < .01

*** *p* < .001.

### 3.4 NPP loss of moderate and extreme dry events

**FIGURE 5:**
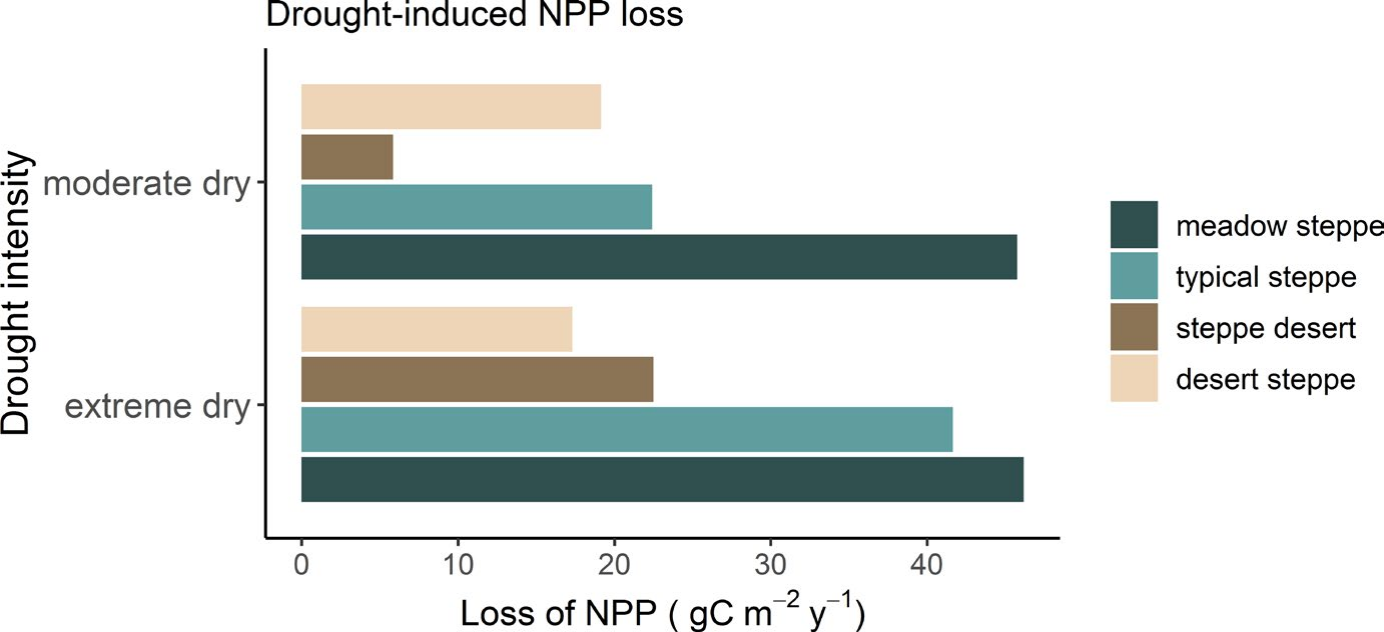
Net primary productivity (NPP) loss of moderate and extreme dry climates in four grassland types

In order to investigate the impacts of moderate and extreme dry events on annual NPP, we assessed the quantitative impacts of these dry events on NPP in four grassland types. Compared with other grasslands, the loss of NPP was highest in meadow steppe both for moderate dry (45.75 gC m^−2 ^year^−1^) and extreme dry (46.18 gC m^−2 ^year^−1^) events (Figure 5). For the typical steppe, the NPP loss caused by moderate dry and extreme dry was 22.42 and 41.65 gC m^−2 ^year^−1^, respectively (Figure 5). Steppe desert showed lower NPP loss in moderate dry events (5.85 gC m^−2 ^year^−1^), but the NPP loss resulted from extreme dry events (22.50 gC m^−2 ^year^−1^) in this grassland was 4 times higher than that in moderate dry events (Figure 5). For desert steppe, NPP loss in both categories of dry events is almost the same (19.16 gC m^−2 ^year^−1^ in moderate dry, and 17.32 gC m^−2 ^year^−1^ in extreme dry). Overall, NPP loss in typical steppe and steppe desert was increased with increasing drought intensity, while NPP loss in meadow steppe and desert steppe was not changed by drought intensity (Figure 5).

## 4 DISCUSSION

Grassland NPP, an important indicator to monitor vegetation health is used to evaluate the functioning and stability of grassland ecosystem (Zhang et al., 2017), and plays an important role in global carbon balance (Sun et al., 2016). However, in recent years, climate extremes posed a serious threat to grassland biodiversity and thus declined grassland productivity (Hossain & Li, 2021a; Isbell et al., 2015; Wilcox et al., 2017; Zhang, Miao, et al., 2020). Given the increasing frequency and intensity of climate extremes, understanding the effects of climate extremes on grassland NPP is an important research goal in ecology and gaining increased importance with the realization that NPP responses to different climate extreme intensities may vary across different grassland types (Lei et al., 2020). In this study, we explored the variations of annual NPP of four grassland types, the responses of NPP to different intensities, and time‐lag of climatic conditions and the loss of NPP resulted from moderate and extreme dry events for the period 2000 to 2019. Our study provides evidence of the differential responses of four grassland types to different intensities and directions of climate extremes, which has important implications in sustainable grassland management under increasing climate extremes.

### 4.1 Variations of NPP

Grassland annual NPP varied significantly across the four grassland types, with a higher annual average NPP in meadow steppe and typical steppe, and a lower annual average NPP in steppe desert and desert steppe (Figure 1a). Similar results were also reported for the spatial distribution of annual NPP in grasslands in the Gansu Province (Zhang, Liu, et al., 2020), where NPP in the southeast (Qilian Mountain, Longnan Mountain, and Gannan Plateau) which is mostly occupied by meadow and typical steppe was highest and NPP in the northwest (Hexi Corridor and North Mountain) which is occupied by steppe desert and desert steppe was lowest (Zhang, Liu, et al., 2020). Similarly, investigating the changes of NPP across different biomes for the period 2000–2010, Liu et al. (2015) also reported that NPP in steppe grasslands was higher than that in desert grasslands, which is consistent with our findings where we found NPP was highest in meadow steppe and lowest in desert steppe. In accordance with our findings, using MODIS NPP product, Liu et al. (2019) assessed NPP variations in Gannan prefecture over 2000–2016 and showed that NPP in alpine meadow was higher than in steppe grassland. Aside from remote sensing‐based NPP observation across different grassland types, consistent with our findings, empirical observations in many grasslands also showed significant difference in the variations of NPP. For example, in diverse vegetation types in northern China, grassland productivity varied significantly among the grasslands occupied by alpine steppe, meadow steppe, alpine meadow, mountain meadow, desert steppe, and typical steppe (Yang et al., 2010). Furthermore, large variations of NPP have been found across grasslands in meadow steppe, desert steppe, meadow, and alpine steppe in the northern Tibetan Plateau (Niu et al., 2019), and grasslands in desert steppe, temperate grassland, meadow steppe, and alpine meadow in Qinghai‐Tibetan Plateau (Dai et al., 2019).

Temporal patterns in NPP in all grasslands in our study showed an increasing trend over the past 20 years (Figure 1b). Consistent with our study, a growing body of evidence also demonstrated an increasing trend of (i) NPP in meadow steppe over 1989–2005, typical steppe over 1980–2006, and desert steppe over 1982–2006 in Inner Mongolia (Lei et al., 2020), and (ii) aboveground biomass in meadow steppe, desert steppe, and steppe desert grasslands in Inner Mongolia over 2005–2012 (Zhao et al., 2014). However, in our study, annual NPP in meadow steppe and typical steppe exhibited a decreasing trend for the period 2012–2019. This decreasing trend of annual NPP in these two grasslands may be due to the increase of drought events, which is in accordance with Liu, Zhang, et al. (2021), which showed that decreasing trend of annual NPP in grasslands in Inner Mongolia over 2000–2017 was associated with climatic conditions. This is because the vegetation in meadow steppe and typical steppe are more productive than those in steppe desert and desert steppe, thus NPP in these productive grasslands may respond negatively to warmer climates. In order to investigate the underlying reason for such discrepancy, we assessed the NPP response to climate extremes of different intensities, which is described in Section 4.2.

### 4.2 Effects of climate extremes and time‐lag on NPP

Climate extreme has been identified as one of the most growing threat to the terrestrial ecosystems, especially to the grassland ecosystems (Liang et al., 2018; Zhao & Running, 2010). The increasing frequency and severity of climate extremes would have profound impacts on grassland productivity (IPBES, 2019). Consistent with our findings on the decreasing trend of annual NPP of all grassland types with increasing dry climate (Figure 2), numerous studies also showed negative relationships between NPP and droughts. For example, assessing the SPI and NPP in desert steppe, typical steppe, and meadow steppe, Lei et al. (2020) found decreasing NPP with increasing intensity of droughts (moderate dry, severe dry, and extreme dry). The significant differences in the annual average NPP among the growing‐season climate extreme intensities in all grassland types in our study were also consistent with Pei et al. (2013), which reported that response of grassland NPP to different climate extreme intensities differed significantly, where annual NPP was the lowest in extreme dry and highest in extreme wet events. The observed negative effects of extreme dry climates on annual NPP in all grasslands might be caused by weakening photosynthesis, increase in evapotranspiration, and decrease in soil water (De Boeck et al., 2011; Knapp et al., 2008). The higher annual NPP of all grasslands during wet events suggests that irrespective of grasslands precipitation (either moderate or extreme wet events) enhances NPP, which is in accordance with the previous studies in temperate, alpine, and desert grasslands (Guo et al., 2012; Hossain & Li, 2021b; Wang et al., 2018). Likewise, Chen et al. (2012) reported the positive effects of precipitation on NPP across the grassland ecosystems in the southern United States over the 20th century.

Vegetation in arid climates has adaptive strategies to cope with perturbations (Volder et al., 2010), which we observed for the steppe desert and desert steppe (Figure 3). Compared with normal climates, NPP in these two grasslands did not significantly decline during growing‐season moderate dry, and extreme dry events (Figure 4). This result suggests that vegetation in these grasslands have a higher resistance to drought (Lei et al., 2020), and supports the notion that in resource‐scarce ecosystems plants allocate more photosynthate to root under dry conditions (Dai et al., 2020) and to shoot under wet conditions (Guo et al., 2012).

Like the vegetation response to different intensities and directions of climate extremes, understanding the effects of time‐lag of climate on grassland productivity is also important in order to explore the mechanisms underlying ecosystem‐climate interaction (Zhe & Zhang, 2021). Irrespective of grassland types, we found annual NPP was significantly correlated with climatic conditions of 1‐ and 2‐month time‐lag, which is consistent with Liu, Zhang, et al. (2021), which reported 1‐ to 3‐month lag effects of temperature and precipitation on the NPP of 11 grassland types in Inner Mongolia. Significant interactions of 3‐month time‐lag of climatic conditions with annual NPP of meadow steppe and typical steppe suggests that productive ecosystems (i.e., higher NPP in these two grasslands) suffer most to climatic fluctuations than less productive ecosystems (e.g., lower NPP in desert steppe and steppe desert).

### 4.3 Drought‐induced NPP loss

In order to differentiate the impacts of moderate and extreme dry events on annual NPP, we analyzed the quantitative impacts of these two intensity droughts for all grasslands (Figure 5). Irrespective of grassland types, the response of NPP to growing‐season moderate and extreme dry events was different. The differential effects of different intensities of drought on NPP loss in our studied grasslands are in accordance with other studies across the world (Chen et al., 2012; Lei et al., 2020; Pei et al., 2013; Sun et al., 2016). NPP loss in typical steppe and steppe desert increased from moderate dry to extreme dry events, which is consistent with Lei et al. (2020), which have reported an exponential growth relationship of NPP loss in the order of moderate dry < severe dry < extreme dry. NPP loss caused by moderate dry events in steppe desert was comparatively lower than that of other grasslands, because vegetation in this grassland may able to absorb mild shocks and thus has higher resistance to moderate dry events. However, NPP loss in this grassland results from growing‐season extreme dry events was four time greater than that of moderate dry events, which suggests that excessive stress has profound impacts on NPP in arid grasslands (Kahmen et al., 2005). An explanation is that persistent extreme dry events stimulate evapotranspiration and lower the photosynthetic rate and rain use efficiency of vegetation in steppe desert (McDowell et al., 2008). Likewise, for typical steppe, NPP loss caused by growing‐season extreme dry was twice than that of moderate dry events, which highlights that vegetation in this moderately productive grassland are also of the great threat of extreme dry events.

Surprisingly, NPP loss in meadow steppe and desert steppe did not increase with increasing intensity of the events from moderate to extreme dry. Different grasslands have different response mechanisms to climate extremes (Hossain & Li, 2021c), some species are very sensitive to perturbations (Tilman et al., 2001), some are resistant to climate extremes (Hossain & Li, 2021b; Isbell et al., 2015), while some are benefitted from the changes (Hector et al., 2010). Vegetation in meadow steppe is very sensitive to climate extremes, thus a small increase in aridity had substantial loss of NPP in this grassland. But as drought intensity increased, plants in meadow steppe either increased their resistance (Isbell et al., 2015) or increased functional compensations among species (Hossain & Beierkuhnlein, 2018). It is expected that desert steppe is less affected by climate extremes, as plants in this grassland are less productive and thus maintain diverse mechanisms (e.g., biomass partitioning) to address extreme droughts (Volder et al., 2010).

Although our study considered the effects of moderate and extreme climatic events and time‐lag effects on annual NPP of meadow steppe, typical steppe, steppe desert, and desert steppe in Inner Mongolia, it is important to investigate the response of these grasslands to other kind of climate extremes (e.g., heat wave). Despite the evidence of substantial loss and gain of annual NPP in our studied grasslands caused by extreme dry and extreme wet events, the examination of resistance and resilience (two most important facets of ecological stability) of the productivity of these grasslands is of importance in sustainable management of grassland ecosystems in the face of climate change.

## 5 CONCLUSIONS

The patterns of grassland annual NPP and their response to climate extremes were investigated in meadow steppe, typical steppe, steppe desert, and desert steppe in Inner Mongolia, China for the period 2000–2019. The main conclusions included:

- Annual NPP varied significantly across grassland types and showed an increasing trend in the order of desert steppe > steppe desert > typical steppe > meadow steppe over the past 20 years. Although NPP in all grassland types showed positive trends, NPP in meadow steppe and typical steppe exhibited a declining trend for the period 2012–2019. The 1‐ and 2‐month time‐lag of climatic conditions had significant effects on annul NPP in all grassland types.
- Growing‐season climate has been found the strong predictor of annual NPP than the annual climate. Annual NPP significantly varied among growing‐season climate extreme intensities, of which loss (gain) of annual NPP was exhibited for dry (wet) climates in all grassland types.
- When the quantitative impacts of extreme and moderate dry events on annual NPP were considered, it is apparent that typical steppe and steppe desert grasslands are of great threat to increasing intensity of drought (i.e., moderate to extreme dry) and meadow steppe, and desert steppe grasslands are sensitive to low‐intensity drought.

This study highlights the key insight into the response of grassland NPP to climatic conditions (moderate and extreme events, and time‐lag) and how the loss of NPP resulted from increasing drought intensity. These findings have important implications for advancing our understanding of how grasslands respond to climate extremes, which is essential for conserving biodiversity, maintaining the functioning and stability of the grassland ecosystem in the face of global climate change. For desert steppe grassland, due to lack of field observation data we did not validate MODIS NPP. Thus, cautions are required to interpret the results of NPP response to climate extremes in desert steppe. Although this study considered MODIS NPP and climatic events of different intensities, future efforts should focus on resistance and resilience of these grasslands based on field observation and other climate extremes.

## CONFLICT OF INTERESTS

The authors declare that they have no competing interests.

## Supporting information

Supplementary Material

## Data Availability

Open access NPP data can be obtained from https://doi.org/10.5067/MODIS/MOD17A3HGF.006. SPEI data can be obtained from http://spei.csic.es/database.html.

## References

[pei310064-bib-0001] Adams, J. M. , Faure, H. , Faure‐Denard, L. , McGlade, J. M. , & Woodward, F. I. (1990). Increase in terrestrial carbon storage from the last glacial maximum to the present. Nature, 348(6303), 711–714.

[pei310064-bib-0002] Allan, E. , Manning, P. , Alt, F. , Binkenstein, J. , Blaser, S. , Blüthgen, N. , Böhm, S. , Grassein, F. , Hölzel, N. , Klaus, V. H. , Kleinebecker, T. , Morris, E. K. , Oelmann, Y. , Prati, D. , Renner, S. C. , Rillig, M. C. , Schaefer, M. , Schloter, M. , Schmitt, B. , … Fischer, M. (2015). Land use intensification alters ecosystem multifunctionality via loss of biodiversity and changes to functional composition. Ecology Letters, 18, 834–843. 10.1111/ele.12469 26096863 PMC4744976

[pei310064-bib-0003] Bao, G. , Chen, J. , Chopping, M. , Bao, Y. , Bayarsaikhan, S. , Dorjsuren, A. , Tuya, A. , Jirigala, B. , & Qin, Z. (2019). Dynamics of net primary productivity on the Mongolian Plateau: Joint regulations of phenology and drought. International Journal of Applied Earth Observation and Geoinformation, 81, 85–97.

[pei310064-bib-0004] Barnes, M. L. , Moran, M. S. , Scott, R. L. , Kolb, T. E. , Ponce‐Campos, G. E. , Moore, D. J. P. , Ross, M. A. , Mitra, B. , & Dore, S. (2016). Vegetation productivity responds to sub‐annual climate conditions across semiarid biomes. Ecosphere, 7(5), e01339. 10.1002/ecs2.1339

[pei310064-bib-0005] Chen, G. , Tian, H. , Zhang, C. , Liu, M. , Ren, W. , Zhu, W. , Chappelka, A. H. , Prior, S. A. , & Lockaby, G. B. (2012). Drought in the southern United States over the 20th century: Variability and its impacts on terrestrial ecosystem productivity and carbon storage. Climatic Change, 114(2), 379–397. 10.1007/s10584-012-0410-z

[pei310064-bib-0006] Cui, A. , Li, J. , Zhou, Q. , Zhu, R. , Liu, H. , Wu, H. , & Li, Q. (2021). Use of a multiscalar GRACE‐based standardized terrestrial water storage index for assessing global hydrological droughts. Journal of Hydrology, 603, 126871.

[pei310064-bib-0007] Dai, L. , Ke, X. , Guo, X. , Du, Y. , Zhang, F. , Li, Y. , Li, Q. , Lin, L. I. , Peng, C. , Shu, K. , & Cao, G. (2019). Responses of biomass allocation across two vegetation types to climate fluctuations in the northern Qinghai‐Tibet Plateau. Ecology and Evolution, 9, 6105–6115. 10.1002/ece3.5194 31161022 PMC6540674

[pei310064-bib-0008] Dai, L. , Xiaowei, G. , Ke, X. , Lan, Y. , Zhang, F. , Li, Y. , Lin, L. , Li, Q. , Cao, G. , Fan, B. , Qian, D. , Zhou, H. , & Du, Y. (2020). Biomass allocation and productivity–richness relationship across four grassland types at the Qinghai Plateau. Ecology and Evolution, 10(1), 506–516. 10.1002/ece3.5920 31988738 PMC6972799

[pei310064-bib-0009] De Boeck, H. J. , Dreesen, F. E. , Janssens, I. A. , & Nijs, I. (2011). Whole system responses of experimental plant communities to climate extremes imposed in different seasons. New Phytologist, 89(3), 806–817. 10.1111/j.1469-8137.2010.03515.x 21054412

[pei310064-bib-0010] de Leeuw, J. , Rizayeva, A. , Namazov, E. , Bayramov, E. , Marshall, M. T. , Etzold, J. , & Neudert, R. (2019). Application of the MODIS MOD 17 net primary production product in grassland carrying capacity assessment. International Journal of Applied Earth Observation and Geoinformation, 78, 66–76. 10.1016/j.jag.2018.09.014

[pei310064-bib-0011] Dong, G. , Zhao, F. , Chen, J. , Qu, L. , Jiang, S. , Chen, J. , & Shao, C. (2021). Divergent forcing of water use efficiency from aridity in two meadows of the Mongolian Plateau. Journal of Hydrology, 593, 125799. 10.1016/j.jhydrol.2020.125799

[pei310064-bib-0012] Guo, Q. , Hu, Z. , Li, S. , Li, X. , Sun, X. , & Yu, G. (2012). Spatial variations in aboveground net primary productivity along a climate gradient in Eurasian temperate grassland: Effects of mean annual precipitation and its seasonal distribution. Global Change Biology, 18(12), 3624–3631. 10.1111/gcb.12010

[pei310064-bib-0013] Hector, A. , Hautier, Y. , Saner, P. , Wacker, L. , Bagchi, R. , Joshi, J. , Scherer‐Lorenzen, M. , Spehn, E. M. , Bazeley‐White, E. , Weilenmann, M. , & Caldeira, M. C. (2010). General stabilizing effects of plant diversity on grassland productivity through population asynchrony and overyielding. Ecology, 91(8), 2213–2220. 20836442 10.1890/09-1162.1

[pei310064-bib-0014] Hossain, M. L. , & Beierkuhnlein, C. (2018). Enhanced aboveground biomass by increased precipitation in a central European grassland. Ecological Processes, 7, 37. 10.1186/s13717-018-0149-1

[pei310064-bib-0015] Hossain, M. L. , & Li, J. (2020). Effects of long‐term climatic variability and harvest frequency on grassland productivity across five ecoregions. Global Ecology and Conservation, 23, e01154.

[pei310064-bib-0016] Hossain, M. L. , & Li, J. (2021a). Biomass partitioning of C_3_‐ and C_4_‐dominated grasslands in response to climatic variability and climate extremes. Environmental Research Letters, 16, 074016. 10.1016/j.scitotenv.2020.143894 33341628

[pei310064-bib-0017] Hossain, M. L. , & Li, J. (2021b). NDVI‐based vegetation dynamics and its resistance and resilience to different intensities of climatic events. Global Ecology and Conservation, 30, e01768. 10.1016/j.gecco.2021.e01768

[pei310064-bib-0018] Hossain, M. L. , & Li, J. (2021c). Disentangling the effects of climatic variability and climate extremes on the belowground biomass of C_3_‐ and C_4_‐dominated grassland across five ecoregions. Science of the Total Environment, 760, 143894. 33341628 10.1016/j.scitotenv.2020.143894

[pei310064-bib-0019] Houghton, R. A. , & Goodale, C. L. (2004). Effects of land‐use change on the carbon balance of terrestrial ecosystems. Geophysical Monograph, 153, 85–98.

[pei310064-bib-0020] IPBES . (2019). Summary for policymakers of the global assessment report on biodiversity and ecosystem services of the Intergovernmental Science‐Policy Platform on Biodiversity and Ecosystem Services. S. Díaz , J. Settele , E. S. Brondízio , H. T. Ngo , M. Guèze , J. Agard , A. Arneth , P. Balvanera , K. A. Brauman , S. H. M. Butchart , K. M. A. Chan , L. A. Garibaldi , K. Ichii , J. Liu , S. M. Subramanian , G. F. Midgley , P. Miloslavich , Z. Molnár , D. Obura , A. Pfaff , S. Polasky , A. Purvis , J. Razzaque , B. Reyers , R. Roy Chowdhury , Y. J. Shin , I. J. Visseren‐Hamakers , K. J. Willis , & C. N. Zayas (Eds.), The global assessment report on biodiversity and ecosystem services. IPBES Secretariat, Bonn, Germany. 56 pp. 10.5281/zenodo.3553579

[pei310064-bib-0021] IPCC . (2013). Climate change 2013: The physical science basis. Contribution of Working Group I to the fifth assessment report of the intergovernmental panel on climate change. Cambridge University Press.

[pei310064-bib-0022] Isbell, F. , Craven, D. , Connolly, J. , Loreau, M. , Schmid, B. , Beierkuhnlein, C. , Bezemer, T. M. , Bonin, C. , Bruelheide, H. , de Luca, E. , Ebeling, A. , Griffin, J. N. , Guo, Q. , Hautier, Y. , Hector, A. , Jentsch, A. , Kreyling, J. , Lanta, V. , Manning, P. , … Eisenhauer, N. (2015). Biodiversity increases the resistance of ecosystem productivity to climate extremes. Nature, 526, 574–577. 10.1038/nature15374 26466564

[pei310064-bib-0023] John, R. , Chen, J. , Giannico, V. , Park, H. , Xiao, J. , Shirkey, G. , Ouyang, Z. , Shao, C. , Lafortezza, R. , & Qi, J. (2018). Grassland canopy cover and aboveground biomass in Mongolia and Inner Mongolia: Spatiotemporal estimates and controlling factors. Remote Sensing of Environment, 213, 34–48. 10.1016/j.rse.2018.05.002

[pei310064-bib-0024] John, R. , Chen, J. , Kim, Y. , Ou‐yang, Z. , Xiao, J. , Park, H. , Shao, C. , Zhang, Y. , Amarjargal, A. , Batkhshig, O. , & Qi, J. (2016). Differentiating anthropogenic modification and precipitation‐driven change on vegetation productivity on the Mongolian Plateau. Landscape Ecology, 31, 547–566. 10.1007/s10980-015-0261-x

[pei310064-bib-0025] Kahmen, A. , Perner, J. , & Buchmann, N. (2005). Diversity dependent productivity in semi‐natural grasslands following climate perturbations. Functional Ecology, 19, 594–601. 10.1111/j.1365-2435.2005.01001.x

[pei310064-bib-0026] Kemp, D. R. , Guodong, H. , Xiangyang, H. , Michalk, D. L. , Fujiang, H. , Jianping, W. , & Yingjun, Z. (2013). Innovative grassland management systems for environmental and livelihood benefits. Proceedings of the National Academy of Sciences of the United States of America, 110(21), 8369–8374. 10.1073/pnas.1208063110 23671092 PMC3666733

[pei310064-bib-0027] Knapp, A. K. , Beier, C. , Briske, D. D. , Classen, A. T. , Luo, Y. , Reichstein, M. , Smith, M. D. , Smith, S. D. , Bell, J. E. , Fay, P. A. , Heisler, J. L. , Leavitt, S. W. , Sherry, R. , Smith, B. , & Weng, E. (2008). Consequences of more extreme precipitation regimes for terrestrial ecosystems. BioScience, 58(9), 811–821. 10.1641/B580908

[pei310064-bib-0028] Kreyling, J. , Beierkuhnlein, C. , Elmer, M. , Pritsch, K. , Radovski, M. , Schloter, M. , Wollecke, J. , & Jentsch, A. (2008). Soil biotic processes remain remarkably stable after 100‐year extreme weather events in experimental grassland and heath. Plant and Soil, 308(s1–2), 175–188. 10.1007/s11104-008-9617-1

[pei310064-bib-0029] Kreyling, J. , Dengler, J. , Walter, J. , Velev, N. , Ugurlu, E. , Sopotlieva, D. , Ransijn, J. , Picon‐Cochard, C. , Nijs, I. , Hernandez, P. , Guler, B. , von Gillhaussen, P. , De Boeck, H. J. , Bloor, J. M. G. , Berwaers, S. , Beierkuhnlein, C. , Arfin Khan, M. A. S. , Apostolova, I. , Altan, Y. , … Jentsch, A. (2017). Species richness effects on grassland recovery from drought depend on community productivity in a multisite experiment. Ecology Letters, 20, 1405–1413. 10.1111/ele.12848 28941071

[pei310064-bib-0030] Lecain, D. R. , Morgan, J. A. , Schuman, G. E. , Reeder, J. D. , & Hart, R. H. (2002). Carbon exchange and species composition of grazed pastures and exclosures in the shortgrass steppe of Colorado. Agriculture, Ecosystems & Environment, 93, 421–435. 10.1016/S0167-8809(01)00290-0

[pei310064-bib-0031] Lei, J. , & Peters, A. J. (2003). Assessing vegetation response to drought in the northern Great Plains using vegetation and drought indices. Remote Sensing of Environment, 87, 85–98. 10.1016/S0034-4257(03)00174-3

[pei310064-bib-0032] Lei, T. , Feng, J. , Lv, J. , Wang, J. , Song, H. , Song, W. , & Gao, X. (2020). Net Primary Productivity Loss under different drought levels in different grassland ecosystems. Journal of Environmental Management, 274, 111144. 10.1016/j.jenvman.2020.111144 32798851

[pei310064-bib-0033] Lei, T. , Wu, J. , Li, X. , Geng, G. , Shao, C. , Zhou, H. , Wang, Q. , & Liu, L. (2015). A new framework for evaluating the impacts of drought on net primary productivity of grassland. Science of the Total Environment, 536, 161–172. 10.1016/j.scitotenv.2015.06.138 26204052

[pei310064-bib-0034] Li, F. , Chen, J. Q. , Zeng, Y. , Wu, B. F. , & Zhang, X. Q. (2018). Renewed estimates of grassland aboveground biomass showing drought impacts. JGR Biogeosciences, 123(1), 138–148. 10.1002/2017JG004255

[pei310064-bib-0035] Li, L. , Zheng, Z. , Biederman, J. A. , Qian, R. , Ran, Q. , Zhang, B. , Xu, C. , Wang, F. , Zhou, S. , Che, R. , Dong, J. , Xu, Z. , Cui, X. , Hao, Y. , & Wang, Y. (2020). Drought and heat wave impacts on grassland carbon cycling across hierarchical levels. Plant, Cell & Environment, 44(7), 2402–2413, 10.1111/pce.13767 32275067

[pei310064-bib-0036] Liang, M. , Chen, J. , Gornish, E. S. , Bai, X. , Li, Z. , & Liang, C. (2018). Grazing effect on grasslands escalated by abnormal precipitations in Inner Mongolia. Ecology and Evolution, 8(16), 8187–8196. 10.1002/ece3.4331 30250694 PMC6144992

[pei310064-bib-0037] Liu, C. Y. , Dong, X. F. , & Liu, Y. Y. (2015). Changes of NPP and their relationship to climate factors based on the transformation of different scales in Gansu, China. Catena, 125, 190–199. 10.1016/j.catena.2014.10.027

[pei310064-bib-0038] Liu, H. , Lin, L. , Wang, H. , Zhang, Z. , Shangguan, Z. , Feng, X. , & He, J.‐S. (2021). Simulating warmer and drier climate increases root production but decreases root decomposition in an alpine grassland on the Tibetan plateau. Plant and Soil, 458, 59–73. 10.1007/s11104-020-04551-y

[pei310064-bib-0039] Liu, H. , Zhang, A. , Liu, C. , Zhao, Y. , Zhao, A. , & Wang, D. (2021). Analysis of the time‐lag effects of climate factors on grassland productivity in Inner Mongolia. Global Ecology and Conservation, 30, e01751. 10.1016/j.gecco.2021.e01751

[pei310064-bib-0040] Liu, J. , Bao‐Ping, M. , Jing, G. , Jin‐Long, G. , Jian‐Peng, Y. , Meng‐Jing, H. , Qi‐Sheng, F. , & Tian‐Gang, L. (2019). Spatio‐temporal dynamic changes of grassland NPP in Gannan prefecture, as determined by the CASA model. Acta Pratacul Sinica, 28(6), 19–32.

[pei310064-bib-0041] Luo, Y. Q. , Zhao, X. Y. , Zuo, X. A. , Li, Y. L. , & Wang, T. (2017). Plant responses to warming and increased precipitation in three categories of dune stabilization in northeastern China. Ecological Research, 4, 1–12. 10.1007/s11284-017-1493-9

[pei310064-bib-0042] McDowell, N. , Pockman, W. T. , Allen, C. D. , Breshears, D. D. , Cobb, N. , Kolb, T. , Plaut, J. , Sperry, J. , West, A. , Williams, D. G. , & Yepez, E. A. (2008). Mechanisms of plant survival and mortality during drought: Why do some plants survive while others succumb to drought? New Phytologist, 178, 719–739. 10.1111/j.1469-8137.2008.02436.x 18422905

[pei310064-bib-0043] Nila, M. S. T. , Beierkuhnlein, C. , Jaeschke, A. , Hoffmann, S. , & Hossain, M. L. (2019). Predicting the effectiveness of protected areas of Natura 2000 under climate change. Ecological Processes, 8, 13. 10.1186/s13717-019-0168-6

[pei310064-bib-0044] Niu, B. , Zeng, C. , Zhang, X. , He, Y. , Shi, P. , Tian, Y. , Feng, Y. , Li, M. , Wang, Z. , Wang, X. , & Cao, Y. (2019). High below‐ground productivity allocation of alpine grasslands on the Northern Tibet. Plants, 8(12), 535. 10.3390/plants8120535 31766615 PMC6963938

[pei310064-bib-0045] Padilla, F. M. , Mommer, L. , de Caluwe, H. , Smit‐Tiekstra, A. E. , Visser, E. J. W. , & de Kroon, H. (2019). Effects of extreme rainfall events are independent of plant species richness in an experimental grassland community. Oecologia, 191, 177–190. 10.1007/s00442-019-04476-z 31401664 PMC6732129

[pei310064-bib-0046] Pei, F. , Li, X. , Liu, X. , & Lao, C. (2013). Assessing the impacts of droughts on net primary productivity in China. Journal of Environmental Management, 114, 362–371. 10.1016/j.jenvman.2012.10.031 23164540

[pei310064-bib-0047] Quan, Q. , Zhang, F. , Meng, C. , Ma, F. , Zhou, Q. , Sun, F. , & Niu, S. (2020). Shifting biomass allocation determines community water use efficiency under climate warming. Environmental Research Letters, 15, 094041. 10.1088/1748-9326/aba472

[pei310064-bib-0048] R Core Team . (2020). R: A language and environment for statistical computing. R Foundation for Statistical Computing. https://www.R‐project.org/

[pei310064-bib-0049] Roxburgh, S. H. , Berry, S. L. , Buckley, T. N. , Barnes, B. , & Roderick, M. L. (2005). What is NPP? Inconsistent accounting of respiratory fluxes in the definition of net primary production. Functional Ecology, 19, 378–382. 10.1111/j.1365-2435.2005.00983.x

[pei310064-bib-0050] Running, S. , & Zhao, M. (2019) MOD17A3HGF MODIS/Terra Net Primary Production Gap‐Filled Yearly L4 Global 500 m SIN Grid V006. NASA EOSDIS Land Processes DAAC. Accessed 2020‐12‐14 from 10.5067/MODIS/MOD17A3HGF.006

[pei310064-bib-0051] Scott, R. L. , Hamerlynck, E. P. , Jenerette, G. D. , Moran, M. S. , & Barron‐Gafford, G. A. (2010). Carbon dioxide exchange in a semidesert grassland through drought‐induced vegetation change. Journal of Geophysical Research, 115, G03026. 10.1029/2010JG001348

[pei310064-bib-0052] Sun, B. , Zhao, H. , & Wang, X. (2016). Effects of drought on net primary productivity: Role of temperature, drought intensity and duration. Chinese Geographical Science, 26(2), 270–282.

[pei310064-bib-0053] Tian, L. , Chen, J. , & Zhang, Y. (2017). Growing season carries stronger contributions to albedo dynamics on the Tibetan plateau. PLoS One, 12(9), e0180559. 10.1371/journal.pone.0180559 28886037 PMC5590739

[pei310064-bib-0054] Tilman, D. , Reich, P. B. , Knops, J. , Wedin, D. , Mielke, T. , & Lehman, C. (2001). Diversity and productivity in a long‐term grassland experiment. Science, 294, 843–845. 10.1126/science.1060391 11679667

[pei310064-bib-0055] Turner, D. , Ritts, D. , Cohen, W. , Gower, S. , Running, S. , Zhao, M. , Costa, M. , Kirschbaum, A. , Ham, J. , Saleska, S. , & Ahl, D. (2006). Evaluation of MODIS NPP and GPP products across multiple biomes. Remote Sensing of Environment, 102, 282–292. 10.1016/j.rse.2006.02.017

[pei310064-bib-0056] Turner, D. P. , Ritts, W. D. , Cohen, W. B. , Maeirsperger, T. K. , Gower, S. T. , Kirschbaum, A. A. , Running, S. W. , Zhao, M. , Wofsy, S. C. , Dunn, A. L. , Law, B. E. , Campbell, J. L. , Oechel, W. C. , Kwon, H. J. , Meyers, T. P. , Small, E. E. , Kurc, S. A. , & Gamon, J. A. (2005). Site‐level evaluation of satellite‐based global terrestrial gross primary production and net primary production monitoring. Global Change Biology, 11(4), 666–684. 10.1111/j.1365-2486.2005.00936.x

[pei310064-bib-0057] van der Werf, G. R. , Randerson, J. T. , Giglio, L. , Collatz, G. J. , Mu, M. , Kasibhatla, P. S. , Morton, D. C. , DeFries, R. S. , Jin, Y. , & van Leeuwen, T. T. (2010). Global fire emissions and the contribution of deforestation, savanna, forest, agriculture, and peat fires (1997–2009). Atmospheric Chemistry and Physics, 10, 11707–11735.

[pei310064-bib-0058] Vicente‐Serrano, S. M. , Beguería, S. , & López‐Moreno, J. I. (2010). A multi‐scalar drought index sensitive to global warming: The Standardized Precipitation Evapotranspiration Index – SPEI. Journal of Climatology, 23(7), 1696–1718. 10.1175/2009JCLI2909.1

[pei310064-bib-0059] Vicente‐Serrano, S. M. , Beguerıa, S. , Lorenzo‐Lacruz, J. , Camarero, J. J. , Lopez‐Moreno, J. I. , Azorin‐Molina, C. , Revuelto, J. , Moran‐Tejeda, E. , & Sanchez‐Lorenzo, A. (2012). Performance of drought ındices for ecological, agricultural and hydrological applications. Earth Interactions, 16(10), 1–27.

[pei310064-bib-0060] Vicente‐Serrano, S. M. , Gouveia, C. , Camarero, J. J. , Beguería, S. , Trigo, R. , Lopez‐Moreno, J. I. , Azorín‐Molina, C. , Pasho, E. , Lorenzo‐Lacruz, J. , Revuelto, J. , Moran‐Tejeda, E. , & Sanchez‐Lorenzo, A. (2013). Response of vegetation to drought time‐scales across global land biomes. Proceedings of the National Academy of Sciences of the United States of America, 110(1), 52–57. 10.1073/pnas.1207068110 23248309 PMC3538253

[pei310064-bib-0061] Volder, A. , Tjoelker, M. G. , & Briske, D. D. (2010). Contrasting physiological responsiveness of establishing trees and a C4 grass to rainfall event, intensified summer drought and warming in oak savanna. Global Change Biology, 16(12), 3349–3362.

[pei310064-bib-0062] Wang, Q. , Yang, Y. , Liu, Y. , Tong, L. , Zhang, Q. P. , & Li, J. (2019). Assessing the impacts of drought on grassland net primary production at the global scale. Scientific Reports, 9, 14041. 10.1038/s41598-019-50584-4 31575904 PMC6773702

[pei310064-bib-0063] Wang, Y. , Chen, J. , Zhou, G. , Shao, C. , Chen, J. , Wang, Y. , & Song, J. (2018). Predominance of precipitation event controls ecosystem CO_2_ exchange in an Inner Mongolian desert grassland, China. Journal of Cleaner Production, 197, 781–793. 10.1016/j.jclepro.2018.06.107

[pei310064-bib-0064] Wang, Y. , Zhang, C. , Meng, F.‐R. , Bourque, C.‐P.‐A. , & Zhang, C. (2020). Evaluation of the suitability of six drought indices in naturally growing, transitional vegetation zones in Inner Mongolia (China). PLoS One, 15(5), e0233525. 10.1371/journal.pone.0233525 32470003 PMC7259598

[pei310064-bib-0065] Wilcox, K. R. , Shi, Z. , Gherardi, L. A. , Lemoine, N. P. , Koerner, S. E. , Hoover, D. L. , Bork, E. , Byrne, K. M. , Cahill, J. Jr , Collins, S. L. , Evans, S. , Gilgen, A. K. , Holub, P. , Jiang, L. , Knapp, A. K. , LeCain, D. , Liang, J. , Garcia‐Palacios, P. , Penuelas, J. , … Luo, Y. (2017). Asymmetric responses of primary productivity to precipitation extremes: A synthesis of grassland precipitation manipulation experiments. Global Change Biology, 23(10), 4376–4385. 10.1111/gcb.13706 28370946

[pei310064-bib-0066] Xu, B. , Yang, X. C. , Tao, W. G. , Qin, Z. H. , Liu, H. Q. , & Miao, J. M. (2007). Remote the grass production in China. Acta Ecologica Sinica, 27, 405–413.

[pei310064-bib-0067] Xu, M. , Peng, F. , You, Q. , Guo, J. , Tian, X. , Xue, X. , & Liu, M. (2015). Year‐round warming and autumnal clipping lead to downward transport of root biomass, carbon and total nitrogen in soil of an alpine meadow. Environmental and Experimental Botany, 109, 54–62. 10.1016/j.envexpbot.2014.07.012

[pei310064-bib-0068] Yang, Y. , Fang, J. , Ma, W. , Guo, D. , & Mohammat, A. (2010). Large‐scale pattern of biomass partitioning across China’s grasslands. Global Ecology and Biogeography, 19, 268–277. 10.1111/j.1466-8238.2009.00502.x

[pei310064-bib-0069] Zhang, B. , Cadotte, M. W. , Chen, S. , Tan, X. , You, C. , Ren, T. , Chen, M. , Wang, S. , Li, W. , Chu, C. , Jiang, L. , Bai, Y. , Huang, J. , & Han, X. (2019). Plants alter their vertical root distribution rather than biomass allocation in response to changing precipitation. Ecology, 100(11), e02828. 10.1002/ecy.2828 31323118

[pei310064-bib-0070] Zhang, F. , Quan, Q. , Song, B. , Sun, J. , Chen, Y. , Zhou, Q. , & Niu, S. (2017). Net primary productivity and its partitioning in response to precipitation gradient in an alpine meadow. Scientific Reports, 7, 15193. 10.1038/s41598-017-15580-6 29123194 PMC5680179

[pei310064-bib-0071] Zhang, J. , Miao, Y. , Zhang, T. , Wei, Y. , Qiao, X. , Miao, R. , Wang, D. , Han, S. , & Yang, Z. (2020). Drought timing and primary productivity in a semiarid grassland. Land Degradation & Development, 31(15), 2185–2195. 10.1002/ldr.3603

[pei310064-bib-0072] Zhang, L. , Ameca, E. I. , Cowlishaw, G. , Pettorelli, N. , Foden, W. , & Mace, G. M. (2019). Global assessment of primate vulnerability to extreme climatic events. Nature Climate Change, 9, 554–561. 10.1038/s41558-019-0508-7

[pei310064-bib-0073] Zhang, M. , Liu, X. , Nazieh, S. , Wang, X. , Nkrumah, T. , & Hong, S. (2020). Spatiotemporal distribution of grassland NPP in Gansu Province, China from 1982 to 2011 and its impact factors. PLoS One, 15(11), e0242609. 10.1371/journal.pone.0242609 33227005 PMC7682893

[pei310064-bib-0074] Zhao, F. , Xu, B. , Yang, X. , Jin, Y. , Li, J. , Xia, L. , Chen, S. , & Ma, H. (2014). Remote sensing estimates of grassland aboveground biomass based on MODIS Net Primary Productivity (NPP): A case study in the Xilingol grassland of northern China. Remote Sensing, 6, 5368–5386. 10.3390/rs6065368

[pei310064-bib-0075] Zhao, M. , & Running, S. W. (2010). Drought‐induced reduction in global terrestrial net primary production from 2000 through 2009. Science, 329(5994), 940–943. 10.1126/science.1192666 20724633

[pei310064-bib-0076] Zhe, M. , & Zhang, X. (2021). Time‐lag effects of NDVI to climate change in the Yamzhog Yumco Basin, South Tibet. Ecological Indicators, 124, 107431.

[pei310064-bib-0077] Zhou, W. , Yang, H. , Zhou, L. , Chen, Y. , Huang, L. , & Ju, W. (2018). Dynamics of grassland carbon sequestration and its coupling relation with hydrothermal factor of Inner Mongolia. Ecological Indicators, 95, 1–11. 10.1016/j.ecolind.2018.07.008

