# Supplementary material for: Response of grassland net primary productivity to dry and wet climatic events in four grassland types in Inner Mongolia
Source: Plant Environ Interact. 2021 Oct 7;2(5):250–62. doi: 10.1002/pei3.10064 (PMC10168099; doi:10.1002/pei3.10064)
Supplement: Supplementary file 1 — Supplementary Material [file PEI3-2-250-s001.pdf]

Supporting information for

Response of grassland net primary productivity to dry and wet climatic events in four grassland types in Inner Mongolia

Md Lokman Hossain[1,2,*], Md. Humayain Kabir [3,4], Mst. Umme Salma Nila[5] Ashik Rubaiyat[6]

[1]Department of Environment Protection Technology, German University Bangladesh, Gazipur, Bangladesh

[2]Department of Geography, Hong Kong Baptist University, Hong Kong

[3]Institute of Forestry and Environmental Sciences, University of Chittagong, Bangladesh

[4]Wegener Center for Climate and Global Change, University of Graz, Austria

[5]CEN Centre for Earth System Research and Sustainability, Institute of Geography, University of Hamburg, Germany

[6]Burckhardt Institute, Tropical Silviculture and Forest Ecology, Faculty of Forest Sciences and Forest Ecology, University of Göttingen, Göttingen, Germany

[*]Corresponding author's email: lokmanbbd@gmail.com

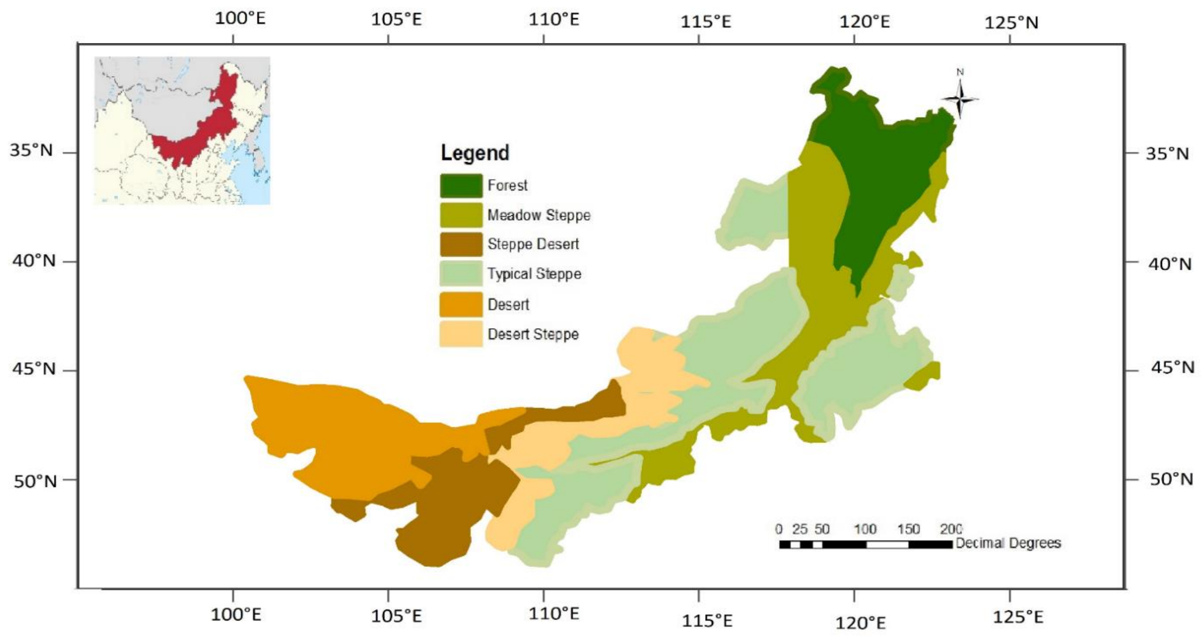

Fig. S1 Locations of four grassland types (meadow steppe, typical steppe, steppe desert, and desert steppe) in the Inner Mongolia, China.

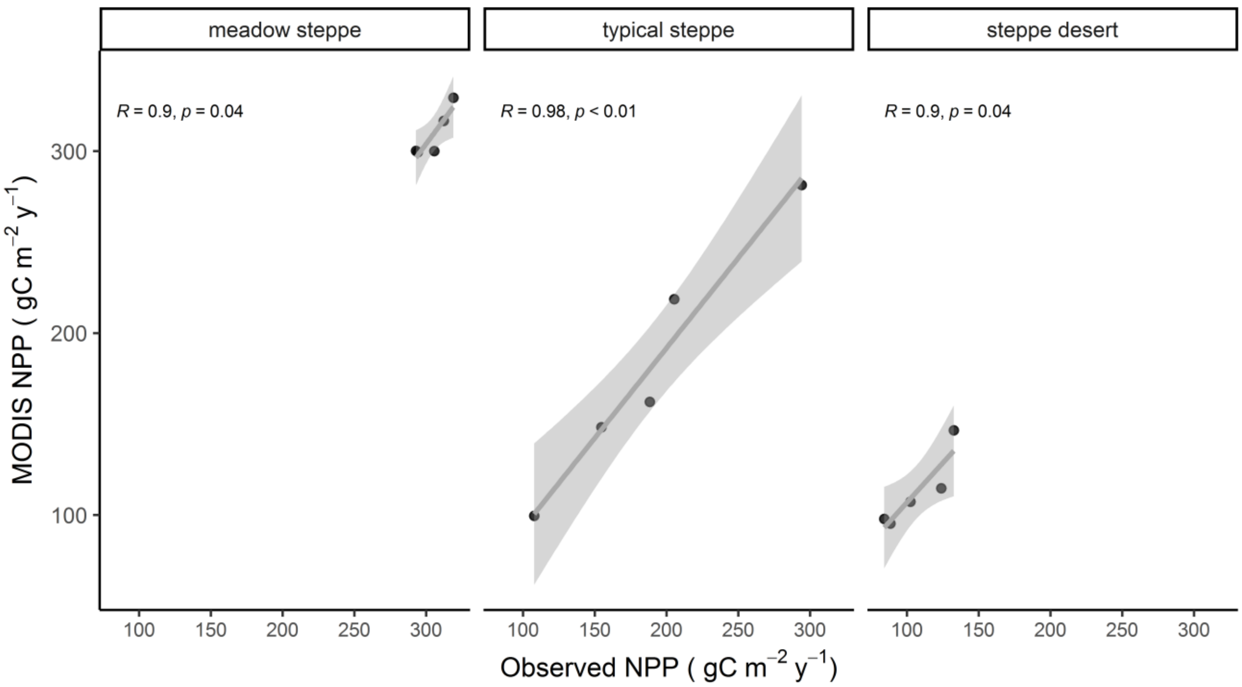

Fig. S2 Correlation between observed net primary productivity (NPP) and MODIS NPP in selected sites in meadow steppe, typical steppe, and steppe desert for the year 2018. Solid lines represent linear regressions of correlation between observed NPP and MODIS NPP. Bands near the lines indicate 95% confidence intervals of correlation of observed NPP with MODIS NPP in selected locations of each grassland type. The *R* and *p* values are the Kendall's correlation coefficient and significance of the correlation, respectively.

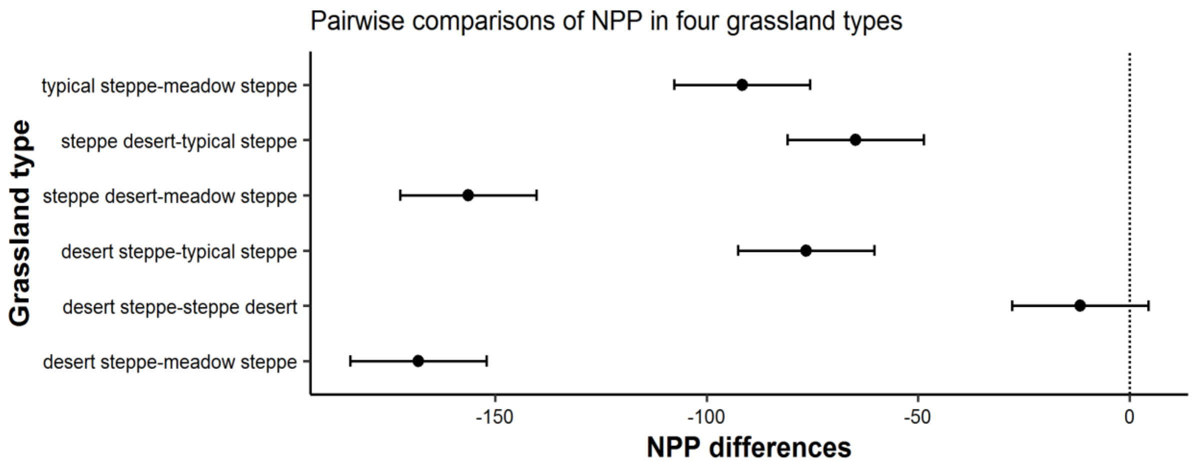

Fig. S3 Pairwise comparisons of net primary productivity (NPP) values in four grassland types. NPP differences between grasslands were observed with post-hoc Tukey's HSD test. The confidence intervals that do not contain 0 represent the significant difference in the pairs. The *p* values of the multiple pairwise comparisons of NPP are shown in Table S2.

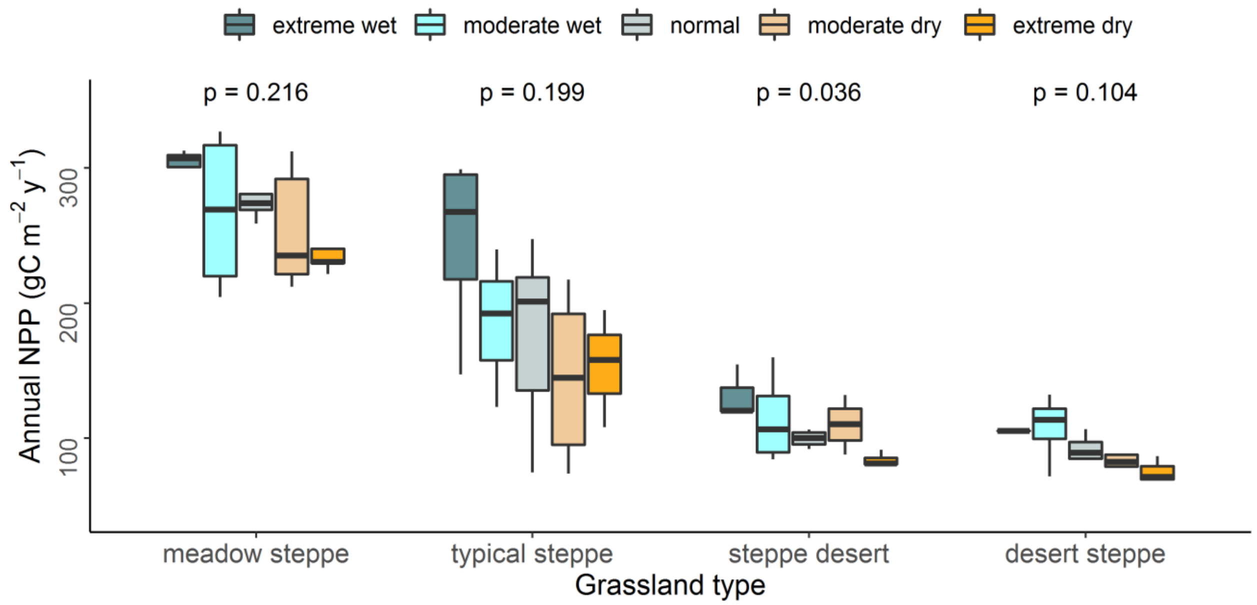

Fig. S4 The response of annual net primary productivity (NPP) to annual climate event intensities (extreme wet, moderate wet, normal, moderate dry, and extreme dry) in four grassland types (i.e. meadow steppe, typical steppe, steppe desert, and desert steppe). Anova *p* indicates a significant difference in the mean NPP among the annual climate event intensities in respective grassland type. Boxes represent the first and third quartiles, solid horizontal lines in the boxes are the medians, and whiskers in the boxes denote the 95% confidence intervals of annual NPP response to each climatic event.

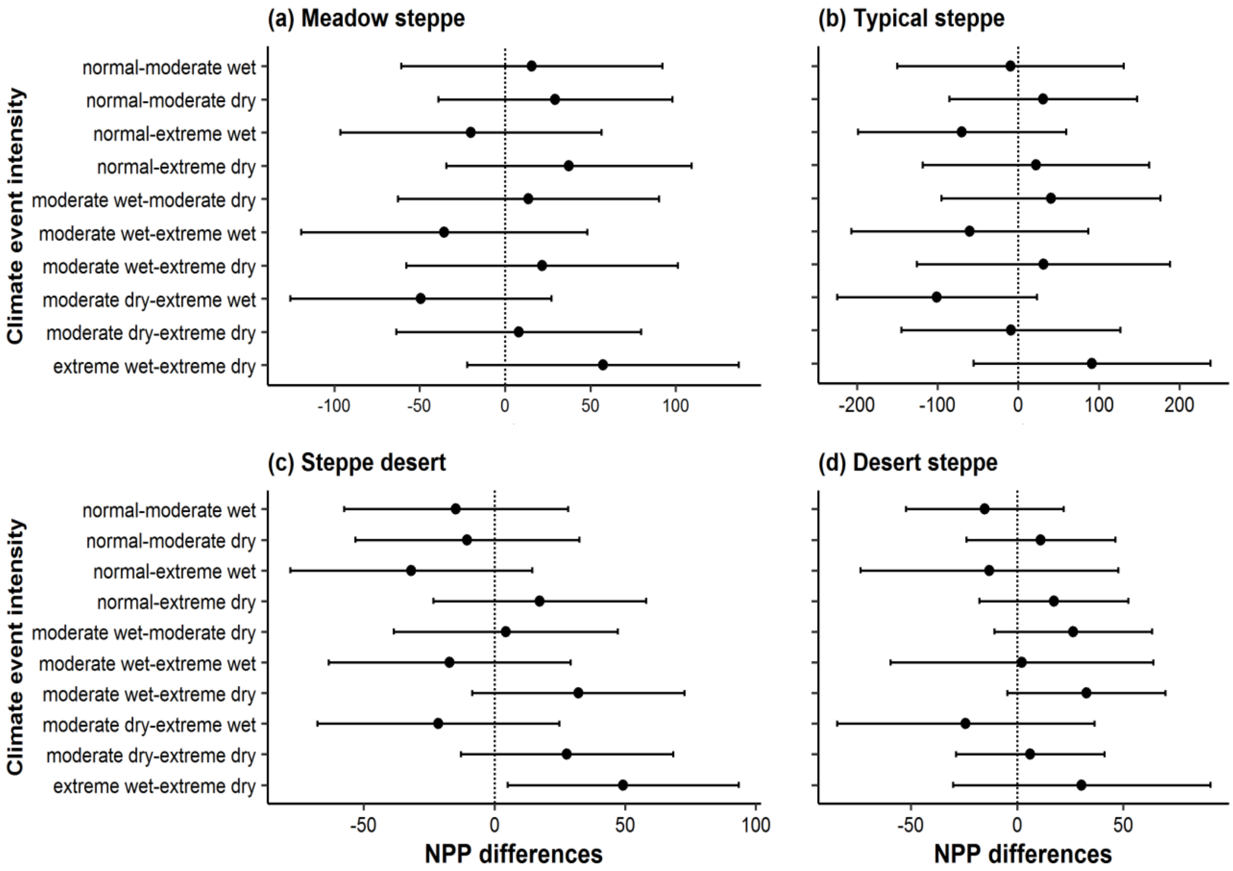

Fig. S5 Pairwise comparisons of annual net primary productivity (NPP) values between the annual climate event intensities for meadow steppe (a), typical steppe (b), steppe desert (c), and desert steppe (d). NPP differences were observed with post-hoc Tukey's HSD test. The confidence intervals that do not contain 0 represent the significant difference in the pairs.

Table S1 Classification of the SPEI values and climate extreme intensity (Isbell et al. 2015).

Climate extreme intensity	SPEI Index value
Extreme wet	≥ 1.28
Moderate wet	0.67 to < 1.28
Normal	-0.67 to < 0.67
Moderate dry	-1.28 to < - 0.67
Extreme dry	≤ - 1.28

Table S2 Pairwise comparisons of NPP values between the grassland types. The NPP differences between each pair were given and the corresponding p values of the comparisons were obtained using post-hoc Tukey's HSD test.

Grassland type	NPP difference	*p* value
typical steppe-meadow steppe	-91.62	< 0.001 ***
steppe desert-meadow steppe	-156.38	< 0.001 ***
desert steppe-meadow steppe	-168.10	< 0.001 ***
steppe desert-typical steppe	-64.75	< 0.001 ***
desert steppe-typical steppe	-76.48	< 0.001 ***
desert steppe-steppe desert	-11.72	0.238

* $p < 0.05$, ** $p < 0.01$, *** $p < 0.001$

Reference

Isbell F, Craven D, Connolly J, Loreau M, Schmid B, Beierkuhnlein C, Bezemer TM, Bonin C, Bruelheide H, de Luca E, Ebeling A, Griffin JN, Guo Q, Hautier Y, Hector A, Jentsch A, Kreyling J, Lanta V, Manning P, Meyer ST, Mori AS, Naeem S, Niklaus PA, Polley HW, Reich PB, Roscher C, Sealoom EW, Smith MD, Thakur MP, Tilman D, Tracy BF, van der Putten WH, van Ruijven J, Weigelt A, Weisser WW, Wilsey B, Eisenhauer N (2015) Biodiversity increases the resistance of ecosystem productivity to climate extremes. Nature 526:574-577.
